# The effect of ethyl alcohol on the severity of injuries in fatal pedestrian victims of traffic crashes

**DOI:** 10.1371/journal.pone.0221749

**Published:** 2019-09-10

**Authors:** Dorota Lasota, Mariusz Goniewicz, Dariusz Kosson, Andrzej Ochal, Paweł Krajewski, Sylwia Tarka, Krzysztof Goniewicz, Dagmara Mirowska-Guzel

**Affiliations:** 1 Department of Experimental and Clinical Pharmacology, Medical University of Warsaw, Warsaw, Poland; 2 Department of Emergency Medicine, Medical University of Lublin, Lublin, Poland; 3 Department of Anesthesiology and Intensive Care, Division of Teaching, Medical University of Warsaw, Warsaw, Poland; 4 Department of Trauma Surgery and Emergency Medicine, Medical University of Lublin, Lublin, Poland; 5 Department of Forensic Medicine, Medical University of Warsaw, Warsaw, Poland; 6 Department of Security Studies, Polish Air Force Academy, Dęblin, Poland; Tongii University, CHINA

## Abstract

**Introduction:**

A substantial percentage of traffic crashes involve people under the influence of ethyl alcohol. In such circumstances, we speak of the possible effect of ethanol upon trauma outcomes. The present research aimed to assess the state of sobriety fatal pedestrian victims and the correlation between the level of sobriety and the severity of injuries.

**Research material and method:**

The data was obtained from the Warsaw Medical University’s Department of Forensic Medicine. The analysis covered the data for the period of 2009–2013; it encompassed 158 fatal pedestrian victims hit by passenger cars. The appropriate methods of statistical analysis were applied.

**Results:**

The majority of the fatal pedestrian victims were individuals under the influence of ethyl alcohol (72.15%). Significant correlations were observed between the concentration of ethyl alcohol and the victims’ gender (p<0.0001) and age (p = 0.0026). The analysis showed that pedestrians under the influence of ethyl alcohol more often died on the scene (78.95%).

**Conclusions:**

Pedestrians under the influence of ethyl alcohol are a significant group of victims of traffic crashes. Ethyl alcohol is not an independent factor affecting the severity of injuries. A higher percentage of pedestrian victims die on the scene, especially in rural areas.

## Introduction

Road traffic crashes are a major social, medical and civilization-related problem. They are one of the leading causes of injuries whose effects may result in permanent impairment of health or even death. In the majority of cases, victims of traffic crashes die of multiple and multi-organ injuries. Furthermore, the gender and age of victims are identified as factors affecting the risk of death in road traffic crashes. In more than 50% of traffic crashes, the fatal victims are people aged 15 to 44. According to the report on health status in Poland, compiled by the National Institute of Public Health, death risk for men is several times as high as for women (the standardized rate for men is 12.4 versus 3.3 for women). Also according to the World Health Organization (WHO) men are more liable to be involved in traffic crashes than women [1]. One factor which significantly influences the increase in the risk of traffic crashes is ethyl alcohol and consequently, a substantial number of crashes involve people under the influence of ethyl alcohol. In such circumstances we can speak of the possible effect of ethanol upon the injury result, i.e. upon the severity of the injuries sustained. Swearingen and coworkers state that the fact of alcohol consumption before sustaining a traumatic injury is the cause of more severe injuries [2, 3], others emphasize that this result does not directly prove that ethyl alcohol is an independent factor affecting the severity of injuries [2]. Other studies consider the positive effect of ethyl alcohol, pointing out its protective action i.e. milder injuries in people being under the influence of alcohol [4, 5]. There is a greater consensus between scholars about the effect of ethyl alcohol on the severity of head injuries. This effect is described as definitely negative [6,7,8]. Therefore, what becomes particularly important is the assessment of injury severity, taking into account various other risk factors, such as, for example, the type and extent of injuries sustained, physiological parameters, the mechanism of injury, any accompanying diseases or the age of the victim. Such an assessment can be conducted on the basis of numerous injury severity scales, including the ones used in this study, i.e. Life Threat Indicator (LTI) [9, 10], International Classification based Injury Severity Score (ICISS) [11, 12], Injury Severity Score (ISS) [13, 14, 15], and New Injury Severity Score (NISS) [16], which use numerical values to evaluate and assess the consequences of an injury.

## Research objectives

The study aimed to assess the level of sobriety and the scale of this phenomenon among fatal pedestrian victims of traffic crashes as well as to evaluate the correlation between the level of sobriety and the severity of injuries expressed in terms of injury severity scales.

## Material and methods

The data for the analysis was obtained from the Department of Forensic Medicine (DFM), Medical University of Warsaw. The analysis covered the data for the period of 2009–2013. The most numerous group of fatal crash victims in the Warsaw metropolitan area recorded in DFM death registers in the period were pedestrians [17], including pedestrians hit by a passenger car [17, 18]. Taking into account criteria such as the mechanism of the injury and age of the victims (≥18 years old), 158 pedestrian victims hit by a passenger car were the study. Based on results of toxicological tests confirming the concentration of ethyl alcohol in blood, muscle, and vitreous body, the studied population was divided into a studied group, i.e. victims under the influence of alcohol (≥0.2 ‰), n = 114 and control group, i.e. sober victims (0, 0 ‰), n = 86.

For the analysis, the appropriate codes from the International Classification of Diseases (ICD, version ICD-10) [19] were assigned to the trauma mechanism and the causes of death identified based on autopsy reports. Furthermore, numerical values of the severity of individual injuries were assigned to injuries, having been calculated according to the Abbreviated Injury Scale (AIS), version 2005, [20]. The assessment of the severity of injuries was conducted based on numerical scales:

1. Threat Indicator (LTI) which does not limit the number of injuries coded (which prevents the need for selecting the most severe injuries to the body) and takes into account the age of victims and injury mechanism;
2. two variants of the International Classification based Injury Severity Score (ICISS) which take account of 10 and 15 injuries to the body; for this study, they are conventionally referred to as ICISS-10 and ICISS-15 respectively;
3. the Injury Severity Score (ISS), where the assessment is based upon the three most severe injuries to the body in three different body regions;
4. the New Injury Severity Score (NISS), which, unlike the ISS, takes account of the three most severe injuries regardless of the body region, even if they are in the same body region.

In the present study, both the LTI score (which is a modification of the ICISS score) and the ICISS score, were based on the ICD-10 codes, and specifically on the catalogues of risk factors which Nogalski calculated and developed on the basis of the ICD-10 for the population of trauma patients in the Lublin province who were hospitalized between 2003 and 2009 [9, 10]. The ISS and the NISS scores were based on the numerical values expressing the severity of individual injuries, calculated according to the AIS scale. Based on the results obtained, a classification of the severity of injuries (ranges) was developed for each of the scales respectively (Table 1). In the present study, the classification was used solely for description. For analysis, the actual, quantitatively measured values for each scale were applied so that the actual results could be taken into account.

**Table 1:** Classification of injury severity.

Scale	Degrees of injury severity	Value ranges
LTI*	Critical injuries	0–0.249
	Very severe injuries	0.25–0.749
	Medium-severe injuries	0.75–0.899
	Mild injuries	≥0.9
ISS**	Critical injuries	>25
	Severe injuries	16–25
	Moderate injuries	10–15
	Mild injuries	1–9

*identical classification of injury severity was used for the ICISS. Source: own elaboration based on [9]

** identical classification of injury severity was used for the NISS. Source: own elaboration based on [16]

The statistical analysis was conducted, taking account of parameters such as gender, age (divided into ranges), place of death (classified as “on the scene” or “in hospital” [within the first 24 hours of hospitalization]), place of occurrence (i.e. the Warsaw metropolitan area, which was divided into the city of Warsaw, the rural parts of the Warsaw metropolitan area and the urban parts of the Warsaw metropolitan area), severity of injuries according to injury severity scales and concentration of ethyl alcohol (divided into ranges). The assessment of the influence of the risk factors upon the severity of injuries measured according to injury severity scales (the LTI, the ICISS-10, the ICISS-15, the ISS, and the NISS) was performed by applying appropriate statistical methods.

The research project was approved and received a favorable opinion from the Bioethical Committee of the Medical University of Warsaw (No. AKBE/112/14).

### 3.1. The statistical analysis methods applied

Standard descriptive statistics (mean values, standard deviations, medians, and ranges) were used in the analysis. The Shapiro-Wilk test was used to verify the hypothesis that the distribution of the analyzed variables corresponds to the normal distribution. The non-parametric Wilcoxon Rank-Sum test was used for the quantitative variables in the comparison of two groups since the distribution of these variables showed deviations from the normal distribution. The Kruskal-Wallis test was used for the quantitative variables in the comparison of several groups. The correlation analysis for the quantitative variables was based on Spearman’s rank correlation coefficient. The relationships between the qualitative variables were tested in a set of contingency tables by applying a chi-squared test or–when the expected values in the cells of the tables were not high enough–Fisher’s precise test. The value of p<0.05 was considered statistically significant. The acquired data were collected in a Microsoft Excel database of an MS Office 2010. The calculations were made using the SAS v. 14.1. system.

## Results

### 4.1. Gender

As a result of the conducted analysis, it was concluded that the fatal pedestrian victims were predominantly male (71.52%), p<0.0001 (Table 2).

**Table 2:** Characteristics of the studied population.

Characteristics	Studied group n (%) = 114 (72.15)	Control group n (%) = 44 (27.85)	Total n (%) = 158 (100.00)
**Gender n (%)**			
Women	21 (18.42)	24 (54.55)	45 (28.48)
Men	93 (81.58)	20 (45.45)	113 (71.52)
**Age n (%)**			
18–24	9 (7.89)	9 (20.46)	18 (11.39)
25–34	16 (14.04)	5 (11.36)	21 (13.29)
35–44	22 (19.30)	4 (9.09)	26 (16.46)
45–54	32 (28.07)	2 (4.55)	34 (21.52)
55–64	25 (21.93)	12 (27.27)	37 (23.42)
65–74	10 (8.77)	12 (27.27)	22 (13.92)
>74	0 (0.00)	0 (0.00)	0 (0.00)
Mean age for women	44.1±12.4	50.9±19	40.8±16.4
Mean age for men	46.6±14.3	47.6±19.5	46.7±15.3
Mean age (M)	46.1±14	49.4±19	47.0±15.6
Median (Me)	48	57	49.5
**Type of body injury n (%)**			
Multiple body regions	97 (85.09)	40 (90.91)	137 (86.71)
Head and neck	12 (10.52)	4 (9.09)	16 (10.13)
Thorax	3 (2.63)	0 (0.00)	3 (1.90)
Abdomen	1 (0.88)	0 (0.00)	1 (0.63)
Upper limb	0 (0.00)	0 (0.00)	0 (0.00)
Lower limb	1 (0.88)	0 (0.00)	1 (0.63)
**Place of occurrence n (%)**			
Warsaw	29 (25.44)	24 (54.55)	53 (33.54)
Urban areas	24 (21.05)	9 (20.45)	33 (20.89)
Rural areas	61 (53.51)	11 (25.00)	72 (45.57)
**Place of death n (%)**			
On the scene	90 (78.95)	22 (50.00)	112 (70.89)
In hospital	24 (21.05)	22 (50.00)	46 (29.11)
**Ethyl alcohol concentration (‰)**			
Minimum	0.20	0 (0.00)	
Maximum	4.40	0 (0.00)	
Mean (M)	2.00±0.9	0 (0.00)	
Women	1.70±0.9	0 (0.00)	
Men	2.10±0.8	0 (0.00)	

### 4.2. Age

The sober fatal pedestrian victims were statistically significantly older Me = 57 years of age (range: 18–74) than the victims under the influence of alcohol Me = 48 years of age (range: 18–74), p<0.0001. In the studied group, pedestrians aged 45–54 were the most heavily represented (28.07%), whereas the most numerous age groups within the control group consisted of victims aged between 55 and 64 (27.7%) and between 65 and 74 (27.7%), p<0.0001 (Table 2).

### 4.3. Type of injuries

Injuries to multiple body regions were the most common type of injuries in both groups n = 137 (86.71%). Among the victims under the influence of alcohol, they constituted n = 97 (85.09%) while among the sober victims n = 40 (90.91%), p = 0.8993 (Table 2).

### 4.4. Place of occurrence

A significant correlation was observed between a given group and a given place of occurrence. For the studied group, the place of occurrence was predominantly the rural parts of the Warsaw metropolitan area (53.51%), while for the control group it was the city of Warsaw (54.55%), p = 0.0011 (Table 2).

### 4.5. Place of death

A significant correlation was found between a given group and a given place of death. For the studied group, the place of death was predominantly "on the scene" (78.95%). For the control group, 50% of deaths occurred on the scene and the other 50% in hospital, within the first 24 hours of hospitalization, p = 0.0007 (Table 2).

### 4.6. The concentration of ethyl alcohol

The presence of ethyl alcohol was observed in more than 72% of fatal pedestrian victims, p<0.0001. Its concentration was 2.00 ± 0.9 and it was in the range of 0.2–4.4 ‰, with ethanol concentrations in men being statistically higher (2.10 ± 0.8) compared to the women’s concentration (1.70 ± 0.9), p = 0.0333 (Table 2).

Significant correlations were found between the concentration of ethyl alcohol and the gender of the fatal pedestrian victims (p<0.0001). With the increase in alcohol concentration, the number of victims in men is increasing. In women, on the contrary, along with the increase in alcohol concentration, the number decreases (Table 3).

**Table 3:** Distribution of ethyl alcohol concentration by ranges for fatal pedestrian victims, depending on the gender, age, place of occurrence and place of death in the studied group.

Factors	Ethyl alcohol concentration (‰)				
	0	0,2–1,2	1,3–1,9	2,0–2,5	>2,5
**Gender n(%)**					
Women	24 (53.33)	7 (15.56)	7 (15.56)	5 (11.11)	2 (4.44)
Men	20 (17.70)	16 (14.16)	24 (21.24)	26 (23.01)	27 (23.89)
**Age n(%)**					
18–24	9 (50.00)	2 (11.10)	5 (27.78)	1 (5.56)	1 (5.56)
25–34	5 (23.80)	3 (14.29)	3 (14.29)	6 (28.57)	4 (19.05)
35–44	4 (15.39)	5 (19.23)	7 (26.92)	8 (30.77)	2 (7.69)
45–54	2 (5.88)	5 (14.71)	4 (11.77)	11 (32.35)	12 (35.29)
55–64	12 (32.43)	6 (16.22)	9 (24.32)	3 (8.11)	7 (18.92)
65–74	12 (54.54)	2 (9.09)	3 (13.64)	2 (9.09)	3 (13.64)
**Place of occurrence n(%)**					
Warsaw	24 (45.28)	6 (11.32)	8 (15.09)	8 (15.09)	7 (13.22)
Urban areas	9 (27.27)	7 (21.21)	9 (27.27)	5 (15.15)	3 (9.10)
Rural areas	11 (15.28)	10 (13.89)	14 (19.44)	18 (25.00)	19 (26.39)
**Place of death n(%)**					
On the scene	22 (19.64)	15 (13.39)	23 (20.55)	26 (23.21)	26 (23.21)
In hospital	22 (47.83)	8 (17.39)	8 (17.39)	5 (10.87)	3 (6.52)

Significant correlations were found between the concentration of ethyl alcohol and the age of the fatal pedestrian victims (p = 0.0026). Then older the victims, the higher the concentrations of alcohol are observed. The range of the age of 45–54 is the limit of this increase. In the case of pedestrians over 54, more pronounced concentrations of alcohol are observed (Table 3).

Furthermore, a significant correlation was observed between the concentration of ethyl alcohol and the place of occurrence, p = 0.0141. The greatest number of deaths occurred in the rural parts of the Warsaw metropolitan area (45.57%). The majority of these deaths were the pedestrians under the influence of alcohol, whereas only 15.28% of them were sober pedestrians. Taking account of the division of alcohol concentration into ranges, the number of victims in the rural parts of the Warsaw metropolitan area increased together with the increase in the concentration of alcohol in the fatal pedestrian victims (Table 3).

A significant correlation was also found between the concentration of ethyl alcohol and the place of death, p = 0.0018. The pedestrians died on the scene more frequently (70.89%) than in the hospital (29.11%). The number of deaths on the scene went up together with the increase in the concentration of alcohol in the fatal pedestrian victims (Table 3).

### 4.7. Correlations

Correlations in the above-mentioned variables were tested in both the control and the studied group. In the control group, significant correlations between age and results obtained in individual scales were observed, although the strength of these correlations is not large (absolute values of correlation coefficients range from 0.14 to -0.34. Correlations between the scales themselves are also important. Such a combination shows how the values of injuries were similar in particular scales. This allows us to ascertain not only the similarity of results obtained by different scales for individual victims but also the entire structure of injuries in the population under study (Table 4).

**Table 4:** Correlations in the control group.

Spearman’s correlation coefficient, n = 44						
**Age**	**Age**	**ISS**	**NISS**	**ICISS-10**	**ICISS-15**	**LTI**
	1.00000	-0.34734	-0.29368	0.17498	0.16194	0.14734
		0.0209	0.0530	0.2559	0.2936	0.3399
**LTI**	**LTI**	**ICISS-15**	**ICISS-10**	**ISS**	**NISS**	**Age**
	1.00000	0.99930	0.99563	-0.41575	-0.40267	0.14734
		<0.0001	<0.0001	0.0050	0.0067	0.3399
**ICISS-10**	**ICISS-10**	**ICISS-15**	**LTI**	**ISS**	**NISS**	**Age**
	1.00000	0.99676	0.99563	-0.41969	-0.40926	0.17498
		<0.0001	<0.0001	0.0046	0.0058	0.2559
**ICISS-15**	**ICISS-15**	**LTI**	**ICISS-10**	**ISS**	**NISS**	**Age**
	1.00000	0.99930	0.99676	-0.42206	-0.40976	0.16794
		<0.0001	<0.0001	0.0043	0.0057	0.2936
**ISS**	**ISS**	**NISS**	**ICISS-15**	**ICISS-10**	**LTI**	**Age**
	1.00000	0.96083	-0.42206	-0.41969	-0.41575	-0.34734
		<0.0001	0.0043	0.0046	0.0050	0.0209
**NISS**	**NISS**	**ISS**	**ICISS-15**	**ICISS-10**	**LTI**	**Age**
	1.00000	0.96083	-0.40976	-0.40926	-0.40267	-0.29368
		<0.0001	0.0057	0.0058	0.0067	0.0530

In the study group, no significant correlations were found between age and results obtained in individual scales. There was also no correlation between the concentration of ethyl alcohol and the results on the scales. However, correlations between the scales themselves are important, but their strength between the ISS scale and the WZŻ, ICISS-10 and ICISS-15 scales as well as the NISS scale and the WZŻ, ICISS-10 and ICISS-15 scales is weaker than in the control group (Table 5).

**Table 5:** Correlations in the studied group.

Spearman’s correlation coefficient, n = 114							
**Age**	**Age**	**ICISS-10**	**ICISS-15**	**LTI**	**Ethyl alcohol concentration**	**ISS**	**NISS**
	1.00000	0.11316	0.10947	0.10286	0.08568	-0.02739	-0.00906
		0.2306	0.2463	0.2761	0.3647	0.7724	0.9238
**Ethyl alcohol concentration**	**Ethyl alcohol concentration**	**NISS**	**Age**	**LTI**	**ICISS-15**	**ISS**	**ICISS-10**
	1.00000	0.10374	0.08568	-0.06533	-0.06472	0.06135	-0.05818
		0.2720	0.3647	0.4898	0.4939	0.5167	0.5386
**LTI**	**LTI**	**ICISS-15**	**ICISS-10**	**ISS**	**NISS**	**Age**	**Ethyl alcohol concentration**
	1.00000	0.99984	0.99663	-0.34415	-0.31118	0.10286	-0.06533
		<0.0001	<0.0001	0.0002	0.0008	0.2761	0.4898
**ICISS-10**	**ICISS-10**	**ICISS-15**	**LTI**	**ISS**	**NISS**	**Age**	**Ethyl alcohol concentration**
	1.00000	0.99670	0.99663	-0.32624	-0.29215	0.11316	-0.05818
		<0.0001	<0.0001	0.0004	0.0016	0.2306	0.5386
**ICISS-15**	**ICISS-15**	**LTI**	**ICISS-10**	**ISS**	**NISS**	**Age**	**Ethyl alcohol concen** **tration**
	1.00000	0.99984	0.99670	-0.34343	-0.31010	0.10947	-0.06472
		<0.0001	<0.0001	0.0002	0.0008	0.2463	0.4939
**ISS**	**ISS**	**NISS**	**LTI**	**ICISS-15**	**ICISS-10**	**Ethyl alcohol concentration**	**Age**
	1.00000	0.96976	-0.34415	-0.34343	-0.32624	0.06135	-0.02739
		<0.0001	0.0002	0.0002	0.0004	0.5167	0.7724
**NISS**	**NISS**	**ISS**	**LTI**	**ICISS-15**	**ICISS-10**	**Ethyl alcohol concentration**	**Age**
	1.00000	0.96976	-0.31118	-0.31010	-0.29215	0.10374	-0.00906
		<0.0001	0.0008	0.0008	0.0016	0.2720	0.9238

### 4.8. Severity of injuries

Based on the classification of the severity of injuries according to each of the injury severity scales, it was shown that, as before the age correction, almost all the injuries sustained by the fatal pedestrian victims in both the studied and the control groups were critical. There were no mild injuries in the studied material (Tables 6 and 7).

**Table 6:** Minimum and maximum values of the severity of injury according to the applied scales in the test and control groups.

Group	Scales	Minimum	Maximum
**Studied group** **n (%) = 114 (57.00)**	**LTI**	0.015	0.810
	**ICISS-10**	0.023	0.875
	**ICISS-15**	0.017	0.875
	**ISS**	10.0	75.0
	**NISS**	22.0	75.0
**Control group** **n (%) = 44 (43.00)**	**LTI**	0.021	0.393
	**ICISS-10**	0.023	0.432
	**ICISS-15**	0.022	0.432
	**ISS**	14.0	75.0
	**NISS**	22.0	75.0

**Table 7:** The division of severity of injuries assessed in the applied injury severity scales in the test and control groups after age correction.

Degrees of injury severity in groups	Scale				
	LTI	ICISS-10	ICISS-15	ISS	NISS
Studied group n = 114					
**Critical injuries** **n%**	102 (89.47)	99 (86.84)	99 (86.84)	103 (90.35)	113 (99.12)
Very severe injuries* n%	11 (9.65)	13 (11.40)	13 (11.40)	9 (7.89)	1 (0.88)
Medium-severe injuries** n%	1 (0.88)	2 (1.76)	2 (1.76)	2 (1.76)	0 (0.00)
**Mild injuries** **n%**	0 (0.00)	0 (0.00)	0 (0.00)	0(0.00)	0 (0.00)
Control group n = 44					
**Critical injuries** **n%**	41 (93.18)	39 (88.64)	39 (88.64)	42 (95.46)	43 (97.73)
Very severe injuries* n%	3 (6.82)	5 (11.36)	5 (11.36)	1 (2.27)	1 (2.27)
Medium-severe injuries** n%	0 (0.00)	0 (0.00)	0 (0.00)	1 (2.27)	0 (0.00)
**Mild injuries** **n%**	0 (0.00)	0 (0.00)	0 (0.00)	0 (0.00)	0 (0.00)

* severe injuries for ISS, NISS

** moderate injuries for ISS, NISS

The analysis of the correlations between the ranges of the LTI, ICISS and ISS scales and the type of injuries in the fatal pedestrian victims showed that these correlations were significant (p = 0.0001, p = 0.0008, p = 0.0280, respectively). Almost all critical injuries (according to the scales used) were injuries to numerous areas of the body.

A significant correlation was also reported between the ranges of the ISS scale and the place of occurrence, p = 0.0306. According to the ISS, the highest percentage of critical injuries was observed in the rural parts of the Warsaw metropolitan area (46.21%).

The analysis of the correlations between the ranges of the LTI and ISS scales and the place of the fatal pedestrian victims’ deaths also showed that these correlations were significant (p = 0.0276 and p = 0.0229, respectively). According to the LTI and ISS, critical injuries were observed in more than 73% of deaths on the scene.

Since only two of the pedestrians had injuries other than critical ones, no analysis was made for the NISS.

## Discussion

Among the fatal pedestrian victims in the studied material, the gender disproportion was especially pronounced in the studied group, i.e. the group of pedestrians under the influence of ethyl alcohol, in which there were almost four times as many men as women. According to the National Institute of Public Health report of 2015, “the phenomenon of excess mortality due to road crashes in rural areas is distinctive. The standardized mortality rates for deaths caused by road crashes were 16.9 for men and 4.2 for women” [21–24]. The results obtained in the investigations reflect this trend.

Research into the role of ethyl alcohol in road crashes shows that ethyl alcohol is a factor that significantly influences their occurrence and consequences. The most frequently discussed issue is that of drivers’ intoxication [25–28]. Meanwhile, alcohol is a well-documented risk factor for crashes among pedestrians and intoxicated pedestrians constitute a potential risk to themselves and other road users since even small concentrations of consumed ethanol cause impaired visual-motor coordination, delayed reaction time, equilibrium disorders and impairment of the ability to control one’s behavior. According to the legal norms in Poland, a person with blood alcohol concentration [BAC] of, or leading to, from 0.2^0^/_00_ to 0.5^0^/_00_ (or breath alcohol concentration [BrAC] of from 0.1 mg to 0.25 mg of alcohol in 1 dm^3^) is considered to be under the influence of alcohol, while a person with BAC of, or leading to, more than 0.5^0^/_00_ (or BrAC of more than 0.25 mg in 1 dm^3^) is considered to be intoxicated (state of insobriety) [29]. Sadly, the above-mentioned limits were vastly exceeded among the studied fatal pedestrian victims (0.2–4.4^0^/_00_). The comparison between the ranges of ethyl alcohol concentration in the fatal pedestrian victims indicates that alcohol consumption in the fatal pedestrian victims was substantially higher among men than among women. The levels of alcohol concentration increased with the increase in the age of the fatal pedestrian victims, but the age of 54 was the limit of the increase. The increase was especially significant in the rural parts of the Warsaw metropolitan area, where the substantial majority of the pedestrians also died on the scene of the crash. This might be caused, among others, by the greater speed of the moving vehicles or to the inferior quality of pre-hospital care. Intensive prevention programs ought to be implemented in these areas so that the safety of pedestrians will be improved.

As far as the question about the effect of ethyl alcohol on the severity of injuries is concerned, the conducted analysis showed no significant correlation. It only revealed that pedestrians under the influence of alcohol die significantly more frequently on the scene of the crash and the frequency increases together with the increase in the concentration of alcohol. This, however, might not be the result of the severity of injuries although the injury severity scales used in the research (the ISS, the LTI) do indicate that the more severe the injuries, the more frequently pedestrians die on the scene. It appears that the place of occurrence itself might be significant here since the percentage of the fatal pedestrian victims was the highest in the rural parts of the Warsaw metropolitan area (45.57%). They were predominantly pedestrians under the influence of ethyl alcohol, the sober pedestrians accounted for only 15.28% of the victims. The lowest percentage of the fatal pedestrian victims was observed in the urban parts of the Warsaw metropolitan area (20.89%), where the sober pedestrians accounted for 27.27% of the victims; in the city of Warsaw, the sober pedestrians accounted for 45.28% of the victims. It is worth remembering here that in the conducted research the number of the fatal pedestrian victims in the rural parts of the Warsaw metropolitan area increased together with the increase in the concentration of ethyl alcohol. The consumption of alcohol is, obviously, only one of the many risk factors influencing the safety of pedestrians in rural areas, but–as the results of the analysis indicate–a very significant one. The current speed of the vehicle involved in the incident is also important. It is usually the subject of the investigation of the circumstances of the event whose findings are secret. Although it is known that as the vehicle speed increases, the kinetic energy released during the collision increases exponentially [30,31], which increases the risk of death and serious injuries, due to the lack of access to information the actual speed of vehicles involved in the incident could not be the criterion for the impact assessment trauma in the presented study. Probably equally important are poorly lit roads, traffic, time of year or weather conditions.

The calculations concerning the presence of ethyl alcohol in the fatal pedestrian victims of traffic crashes and their age in analysis support the conclusion that the severity of injuries decreases together with the age of victims (correlations), but only in the case of those who were not under the influence of alcohol. According to Brongel, age is a kind of trauma catalyst, affecting each "stage" of trauma. "The less severe the trauma and the older the patient, the greater the influence of age upon the trauma effect. In other words, the older the casualty is, the less severe injury may lead to their death. Therefore, age, like gender, as a risk factor for a crash, is also a »moderator« of its consequences when other demographic factors diminish in importance” [32]. The influence of age upon the outcome of injury treatment is mainly connected with the presence of various age-related diseases as well as with the difference in the response of the organism to trauma, which numerous authors describe and highlight in their works. The influence of ethyl alcohol upon the severity of injuries is an issue widely discussed in the sources. However, the published research results are inconclusive. According to many authors, ethyl alcohol is not an independent factor affecting the severity of injuries. Researchers emphasize the weight of risk factors other than alcohol [2–8]. The conducted analysis leads to analogous conclusions. The obtained results of the research indicate areas of public health that require the implementation of remedial processes. They point to the problem of intoxication among road users in a wider sense. They point to the need to take action to improve safety and eliminate risk factors, which undoubtedly include alcohol, in order not only to reduce the number of crashes but above all their effects, i.e. injuries and deaths [33].

## Conclusions

1. Pedestrians under the influence of alcohol constitute a significant group of victims of road traffic crashes. Educational campaigns focusing on the reduction of ethyl alcohol consumption should be addressed to all road users and not only to drivers.
2. Ethyl alcohol is not an independent factor affecting the severity of injuries. It cannot be conclusively stated that it exerts direct effect upon the severity of injuries in fatal pedestrian victims of traffic crashes.
3. A higher percentage of pedestrian victims die on the scene of the crash in rural areas. Intensive prevention programs should be implemented in these areas to improve the safety of pedestrians as unprotected road users.

## Supporting information

S1 Dataset (XLSX)
